# Supplementary material for: Learning curve and surgical outcome of robotic assisted colorectal surgery with ERAS program
Source: Sci Rep. 2022 Nov 29;12:20566. doi: 10.1038/s41598-022-24665-w (PMC9709162; doi:10.1038/s41598-022-24665-w)
Supplement: Supplementary file 2 — Supplementary Information 2. [file 41598_2022_24665_MOESM2_ESM.zip › RAL Raw data-Ñ[▒K/082/OP N_ñw╝╨░O▒Kñσ.pdf]

W95 / W95

臺中榮民總醫院

手術室護理記錄

[redacted]

## 基本資料

- 室別： 開刀房-17室，手術科別： CRS，手術類別： 常規，日期： 2021/05/13
- 進入時間： 1420 ； 麻醉開始時間： 1435 ； Time Out時間： 1520 ； 劃刀時間： 1521 ； 手術結束時間： 1920 送出時間： 1940 ；
- 麻醉方式： GE

  診斷：RO A COLON CA

  手術名稱：Robot Assisted Colectomy(R)

  手術局部用藥：NIL.

  術前預防性抗生素：
    - 抗生素名稱一： Veterin 1GM IVD由麻醉護理師給予
    - 時間： 1515
    - 上次簽章: 張秀敏

  ，追加預防性抗生素：
    - 抗生素名稱一： Veterin 1GM IVD由麻醉護理師給予
    - 時間： 1915
- 手術醫師： 蔣鋒帆，楊曙榮，古朋育，劉佳樺
- 麻醉醫師： VS 齊光彪
- 
    - 刷手護士A ： 吳婉寧／陳盈妤，時間： 1420，接替刷手一： 張秀敏，時間： 1800
    - 流動護士A ： 張秀敏／陳妍希，時間： 1420，接替流動一： 鄭鈞躍，時間： 1800，接替流動二： 陳妍希，時間： 1830

## 皮膚狀況

A-V Shunt： 無

術前： 完整

記錄者： 上次簽章: 張秀敏

術後： 完整

記錄者： 上次簽章: 陳妍希

## 手術準備

手術臥式 單一手術臥式

- 
    - 手術臥式： 仰臥
    - 使用附件： 溫毯 38 ℃，頭圈，其它： silicone pad x1、頭架
    - 皮膚準備： 10%Aq Betadine，Hibiscrub，2% Chlorhexidine
    - 電燒： 使用電燒: 電極片位置： 左大腿，雙極電燒，記錄者： 上次簽章: 陳妍希

止血帶T： 無

## 植入物

- 植入物名稱/號碼/數量/部位：covidien GIA/80-3.8 x2/colon

，記錄者： 上次簽章: 鄭鈞躍

## 標本送檢

- 細菌培養 無
- 細胞學 無
- 冰凍切片 無
- 病理標本

  組織名稱一：A-COLON

  ，

  組織名稱二：omentum
- 截肢 無

標本交付 標本： 2 袋，標本： 0 盒，標本： 0 瓶，細胞學： 0 管，Culture Tube： 0 管，被交付醫師： CR 楊曙榮，

- 記錄者： 陳妍希
- 時間： 1910

## 傷口術後敷料

傷口敷料： 傷口敷料： 無

- 請於圖上標示部位：

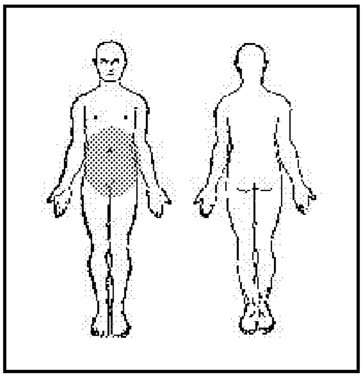

- 傷口敷料： 軟性敷料

## 導管

| 導管 | 天數 | 導管資訊 | 插入日期 | 插入時間 | 更換日期 | 拔除日期 | 拔除時間 | 拔除原因 | 預計拔除/更換 | 預計拔除/更換日期 |
|---|---|---|---|---|---|---|---|---|---|---|
| Foley(1) | 1 | 號碼:16 Fr.; 部位:膀胱; 註記:balloon10c.c.; | 2021/05/13 | 1500 | | | | | | |
| Double Lumen CVC(1) | 1 | 號碼:7 Fr.; 深度:15cm; 部位:jugular (右); 象限:RU; | 2021/05/13 | 1453 | | | | | | |
| Epidural PCA(1) | 1 | 部位:硬脊膜; | 2021/05/13 | 1400 | | | | | | |
| A-line (1) | 1 | 號碼:20 G; 部位:右手; | 2021/05/13 | 1505 | | | | | | |

## 用物計數

- 手術中出血量： 少 c.c.
- 手術中尿量： 300 c.c.
-
    - 其它： 無

用物計數： 多切口同時縫合

- 1.器械： 正確，2.縫針： 正確，3.刀片： 正確，4.紗布： 正確，5.腹部墊： 未使用，6.紗球： 未使用，7.PEANUTS ： 未使用，8.紗條： 未使用，9.絲帶： 未使用，10.針頭： 未使用，11.Vessel loop： 未使用，12.Boots： 未使用，13.Jelco： 未使用，14.棉條： 未使用，
    - 第一次：
        - 刷手護士 ： 張秀敏
        - 流動護士： 上次簽章: 鄭鈞躍
        - 時間： 1825

    ,
    - 第二次：
        - 刷手護士 ： 張秀敏
        - 流動護士： 上次簽章: 陳妍希
        - 時間： 1830

    ,
    - 第三次：
        - 刷手護士 ： 張秀敏
        - 流動護士： 上次簽章: 陳妍希
        - 時間： 1840

## 備註

備註： 達文西30度鏡NO.2

- 記錄者： 上次簽章: 張秀敏
- 時間： 1440

填寫護理人員 陳妍希

正式摘要

W95 / W95

doctorRank:B
